# Gut Microbiota and its Metabolites: Bridge of Dietary Nutrients and Alzheimer’s Disease

**DOI:** 10.1016/j.advnut.2023.04.005

**Published:** 2023-04-17

**Authors:** Guangsu Zhu, Jianxin Zhao, Hao Zhang, Gang Wang, Wei Chen

**Affiliations:** a State Key Laboratory of Food Science and Technology, Jiangnan University, Wuxi, Jiangsu, China; b College of Food Science and Technology, Henan University of Technology, Zhengzhou, Henan, China; c School of Food Science and Technology, Jiangnan University, Wuxi, Jiangsu, China; d (Yangzhou) Institute of Food Biotechnology, Jiangnan University, Yangzhou, Jiangsu, China; e National Engineering Center for Functional Food, Jiangnan University, Wuxi, Jiangsu, China

**Keywords:** Alzheimer’s disease, gut microbiota, microbial metabolites, microbiota–gut–brain axis, dietary interventions

## Abstract

Alzheimer’s disease (AD) is a neurodegenerative disease characterized by progressive cognitive impairment and neuroinflammation. Recent research has revealed the crucial role of gut microbiota and microbial metabolites in modulating AD. However, the mechanisms by which the microbiome and microbial metabolites affect brain function remain poorly understood. Here, we review the literature on changes in the diversity and composition of the gut microbiome in patients with AD and in animal models of AD. We also discuss the latest progress in understanding the pathways by which the gut microbiota and microbial metabolites from the host or diet regulate AD. By understanding the effects of dietary components on brain function, microbiota composition, and microbial metabolites, we examine the potential for manipulation of the gut microbiota through dietary intervention to delay the progression of AD. Although it is challenging to translate our understanding of microbiome-based approaches to dietary guidelines or clinical therapies, these findings provide an attractive target for promoting brain function.

**Statement of Significance**

This review integrates the current evidence and potential mechanisms of gut microbiota and its metabolites in AD and comprehensively reviews the possibility of leveraging dietary interventions to prevent AD progression, with a focus on the gut–microbiota–brain connections and diet–metabolite–host interactions.

## Introduction

Alzheimer’s disease (AD) is a complex condition characterized by the presence of β-amyloid and tau proteins [1]. The deposition of amyloid plaques and neurofibrillary tangles in the brain obstruct normal cognitive function. The symptoms usually manifest as progressive changes in memory, thinking, judgment, behavior, mood, and emotions; eventually, the symptoms become severe enough to affect activities of daily living and social autonomy [2]. The 2021 World Alzheimer Report has stated that over 55 million people live with dementia globally, projected to increase to 78 million by 2030. As one of the costliest chronic diseases, the current annual cost for treatment and health care is estimated to be one trillion dollars, with forecasts predicting a cost of 2 trillion dollars by 2030, reflecting the growing global public health burden. On January 6, 2023, the United States Federal Drug Administration approved the amyloid β peptide (Aβ)-targeting monoclonal antibody, Leqembi (lecanemab-irmb), for the treatment of AD through the accelerated approval pathway [3, 4]. Given that no effective therapeutic approach has been shown to halt the development of AD, disease-modifying interventions may become an important strategy to slow cognitive loss and improve the quality of life [2].

Historically, neurological disorders have been considered to be driven by dysfunctional brain and nervous system development; however, seminal research from the perspective of the gut–brain interaction has indicated that the development of the brain is also affected by the immune and metabolic state of the body [5]. There is considerable research demonstrating that the gut microbiota, as resident bacterial communities in the host, make vital contributions to the formation and functions of the neurological and immune systems as well as metabolism and, via the circulatory system, the development of various organs [6, 7]. At the intersection of neuroscience and microbiology, the gut microbiome is a dynamic entity that can change in composition and structure throughout the host’s lifespan and in response to changing environmental factors, particularly dietary factors [8]. Recent advances in 16S rRNA and shotgun metagenome sequencing technologies and their reduced costs have made it possible to dissect microbiota–gut–brain associations [9]. Importantly, there is evidence of the potential role of the gut microbiota from animal and human studies. Gut microbiota plays a crucial role in the modulation of behavior and brain function in individuals with AD [10, 11].

The gut microbial community generates numerous metabolites, as detected by metabolomic screening [12]. These microbiota-derived metabolites may be released into the gut or transported to many distant organs via the circulatory system and thus exert a diverse range of effects on the intestinal homeostasis and brain health of the host [12]. Recent technologic advancements in metabolomic analyses have made it possible to identify bioactive metabolites. The development of multi-omics techniques has enabled greater insights into their regulatory mechanisms. Specific metabolites closely linked with cognition have been observed in the hippocampus, indicating that these metabolites may serve as active drivers of interactions between the microbiota and the brain [11]. Despite increased awareness of the potential function of the gut microbiome and microbial metabolites in AD, the mechanistic links underlying the gut–brain interaction remain to be elucidated.

As one of the most important environmental factors, dietary changes affect the composition of the gut microbiome [7]. As dietary components act as substrates for gut microbes, the types of foods consumed and the frequency of meals further affect the production of downstream bacterial metabolites [11]. A recently published review discussed the possibility of using different dietary interventions to prevent cognitive decline and reduce the risk of AD [13]. The personal view by Yassine et al. [14] highlights that the effects of dietary nutrition on cognition might be amplified in specific population subgroups, such as APOE genotypes, requiring a personalized approach. Further, a significant amount of evidence has shown that polyphenol treatment can alleviate cognitive defects in mouse models of AD [15]. A recent meta-analysis reviewed 8 clinical intervention studies and concluded that daily probiotic administration can improve cognitive function, particularly in individuals with mild cognitive impairment (MCI) [16]. Dietary fiber, composed of complex plant-derived polysaccharides, is fermented by bacteria into SCFAs, which may subsequently decrease neuroinflammation and improve memory impairment in mouse models of AD [17].

In this review, we consider the possibility of leveraging dietary interventions to prevent AD progression through the regulation of gut microbiota and their metabolites. We begin by reviewing the major taxonomic (mainly bacterial phyla and families) and functional characteristics of AD-related intestinal microbiota in human and animal studies. We then discuss the potential mechanisms by which specific dietary nutrients and foods can affect cognitive function, with a focus on the gut–microbiota–brain connections and diet–metabolite–host interactions. Furthermore, we present examples of microbiome-mediated strategies to improve cognitive function or delay the progression of AD. Finally, we discuss the challenges confronting the development of microbiota-targeted interventions and outline future directions for epidemiological and experimental studies.

## Gut Microbiota and AD

Quantitative and qualitative changes in the gut microbiome are involved in many neurological disorders such as AD, Parkinson’s disease (PD), and autism spectrum disorder [6, 18]. Here, we review the literature on AD-related microbiota components and their potential function in disease development in animal and clinical studies.

### AD-related microbiota in animal models

Indirect evidence from animal studies has contributed to the hypothesis that the gut microbiome is involved in the progression of AD. First, significant gut microbiome alterations have been found in several different animal models of AD [19, 20]. For example, one of the most widely used amyloidosis models in AD-related research, double-transgenic APP/PS1 mice, exhibited microbiota alterations at a young age (3 mo), gradually escalating to more obvious alterations in the prevalence of inflammation-related bacteria at later ages (6 mo and 9 mo) [21]. Daniel et al. [22] evaluated the microbiota structure and cognitive function of APP/PS1 mice and found a sex-dependent difference in gut microbiota composition. Interestingly, male APP/PS1 mice show greater impairments of the gut environment than female mice and a markedly negative correlation with the prevalence of butyrate-producing bacteria. Moreover, a correlation analysis of the microbiota and pathological changes in the brains of 10-mo-old mice revealed that the abundance of 10 bacterial taxa was negatively associated with tau pathology. These findings indicate that the gut microbiota changed in both amyloidosis and tauopathy mouse models. Although these transgenic models have been crucial in demonstrating the role of microbiota in AD, more animal models should be established to further investigate the roles of specific gut microbes in the progression of AD.

Second, short-term antibiotic administration disrupts the overall structure and composition of the gut microbiota. Importantly, fecal microbiota transplantation from 16-mo-old APP/PS1 mice has been shown to increase Aβ accumulation in antibiotic-treated APP/PS1 mice [23]. Third, Aβ pathology has been shown to dramatically increase in the cerebrum of germ-free (GF) mice with AD [19], whereas, in turn, the colonization of microbiota from APP/PS1 mice exacerbates the Aβ pathology of GF APP/PS1 mice. Fourth, transplanting microbiota from young mice (3–4 mo) to aged mice (19–20 mo), selectively attenuated age-associated cognitive impairment and reversed age-associated changes in gut microbiota and hippocampal metabolites [24].

Overall, data from these studies highlight intestinal microbiota's regulatory effects on AD progression. The effects of the gut microbiome on mediating susceptibility and attenuating cognitive and behavioral deficits are only now being understood.

### AD-related microbiota in human studies

Over the past 5 years, many clinical studies have clarified the potential role of gut bacteria-mediated immunity in the progression of MCI or AD (Table). However, cohort studies have only been performed in a few countries such as China and the United States. In 2017, a seminal study in the United States found that patients with AD have decreased microbial diversity, as determined by α-diversity analysis, and significant composition differences, as determined by β-diversity analysis, compared with healthy subjects [25]. A similar decrease in bacterial diversity in patients with AD was found in several subsequent studies [[26], [27], [28]]. However, no significant α-diversity differences were observed in other studies [[29], [30], [31], [32], [33], [34], [35]]. Notably, all of the published cross-sectional studies reported significant differences in the intestinal microbiota composition between patients with AD and healthy individuals, although these studies performed β-diversity analyses using different algorithms such as principal coordinate analysis; non–metric-multidimensional scaling based on weighted/unweighted UniFrac, Bray-Curtis, or Jaccard distances; constrained analysis of principal coordinates; and partial least square-discriminant analysis.

**TABLE:** Alterations in microbial diversity and composition associated with Alzheimer’s diseases (from human cohorts).

Reference	Country	Population	Sequencing methods	Alpha-diversity changes	Beta-diversity changes	Enriched microbiota in AD	Decreased microbiota in AD
(Yıldırım et al., 2022 [36]	Turkish	27 MCI 47 AD 51 Control	16S rRNA amplicon sequencing	1. Shannon & Simpson indices, no significance change; 2. richness index & Chao1 ↓.	PCoA & CAP, distinct separation.	Genus: *Prevotella* & *Bacteroides*.	Genus: *Roseburia*, *Lactobacillus* & *Fusicatenibacter.*
Sheng et al., 2022 [38]	China	11 MCI 11 AD 34 Control	16S rRNA amplicon sequencing	Chao1 & ACE indexes ↓	1. NMDS, significant difference; 2. PCoA, marginal difference.	1. Phylum: Bacteroidetes; 2. Class: Bacteroidia; 3. Order Bacteroidal.	1. Phylum: Firmicutes; 2. Class: Clostridia, Deltaproteobacteria; 3. Order: Clostridiales, Desulfovibrionales; 4. Family: Lachnospiraceae, Desulfovibrionaceae, Ruminococcaceae; 5. Genus: *Bilophila* & *Faecalibacterium.*
Zhou et al., 2021 [35]	China	60 AD 32 Control	16S rRNA amplicon sequencing	1. Observed species ↓; 2. Chao1, ACE & Shannon index, no significant decline.	PCoA & PLS-DA, significant separation.	Genus: *Bifidobacterium*, *Sphingomonas*, *Lactobacillus*, & *Blautia.*	Genus: *Odoribacter*, *Anaerobacterium*, & *Papillibacter*.
Pan et al., 2021 [33]	China	22 MCI 26 Control	16S rRNA amplicon sequencing	Chao, ACE, Shannon, & Simpson indices, no significant difference.	PCoA & NMDS, significant differences.	Species: *Staphylococcus intermedius*.	Species: *Bacteroides salyersiae*.
P. Liu et al., 2021 [32]	China	20 MCI 22 Control	16S rRNA amplicon sequencing	ACE, Chao 1, Simpson's & Shannon indices, no significant difference.	Not reported.	1. Family: Lachnospiraceae; 2. Genus: *Blautia* & *Bacteroides.*	1. Family: Veillonellaceae & Ruminococcaceae; 2. Genus: *Bacteroides*.
Xi et al., 2021 [34]	China	21 AD 44 Control	16S rRNA amplicon sequencing	ACE, Shannon, & Simpson indices, no significant difference.	PCoA, significant differences.	Genus: *Agathobacter*, *Eubacterium_ventriosum_group*, *Lachnospiraceae_NC2004*, *Coprococcus_1*, *Faecalibacterium*, *Ruminococcaceae_UCG-007*, *Alloprevotella*, *Atopobium*, *Parvimonas*, *Cloacibacillus*, *Solobacterium*, *Pseudomonas.*	Genus: *Tyzzerella* & *Erysipelatoclostridium*.
Hou et al., 2021 [31]	China	30 AD 47 Control	16S rRNA amplicon sequencing	Sobs & Shannon index, no significant difference.	PCoA, slight differences.	1. Phylum: Proteobacteria; 2. Order: Enterobacteriales, Deltaproteobacteria & Desulfovibrionales; 3. Family: Enterobacteriaceae & Desulfovibrionaceae; 4. Genus: *Escherichia–Shigella*, *Ruminococcaceae_UCG_002*, *Shuttleworthia*, *Anaerofustis, Morganelia*, *Finegoldia* & *Anaerotruncus.*	1. Family: Enterococcaceae; 2. Genus: *Megamonas*, *Enterococcus*& *Anaerostipes.*
M. Guo et al., 2021 [30]	China	18 AD 20 MCI 18 Control	16S rRNA amplicon sequencing	Evenness, faith pd & Shannon index, no significant difference.	PCA, significant differences.	Genus: *Prevotella.*	Genus: *Bacteroides*, *Lachnospira* & *Ruminiclostridium 9.*
Ling et al., 2021 [28]	China	100 AD 71 Control	16S rRNA amplicon sequencing	ACE, Chao1, Shannon & Simpson indices↓.	PCoA, significant differences.	Genus: lactate-producing *Bifidobacterium.*	Genus: butyrate-producing *Faecalibacterium.*
P. Liu et al., 2019 [37]	China	33 AD 32 MCI 32 Control	16S rRNA amplicon sequencing	1. ACE & Chao 1, no significant difference; 2. Shannon & Simpson indices, ↓.	PCoA, significant differences.	1. Phylum: Proteobacteria; 2. Order: Gammaproteobacteria & Enterobacteriales; 3. Family: Enterobacteriaceae.	Phylum: Firmicutes.
Li et al., 2019 [29]	China	30 AD 30 MCI 30 Control	16S rRNA amplicon sequencing	1. PD whole tree, ↓; 2. Chao1, observed species & Shannon index, no significant differences.	PCA, significant differences.	Genus: *Dorea*, *Lactobacillus*, *Streptococcus*, *Bifidobacterium*, *Blautia* & *Escherichia.*	Genus: *Alistipes*, *Bacteroides*, *Parabacteroides*, *Sutterella* & *Paraprevotella*.
Haran et al., 2019 [40]	USA	24 AD 51 Control	NextSeq500/Metagenomic analysis	Not reported.	tSNE, significant differences.	Genus: *Bacteroides*, *Alistipes*, *Odoribacter* & *Barnesiella.*	Genus: *Lachnoclostridium.*
Zhuang et al., 2018 [26]	China	43 AD 43 Control	16S rRNA amplicon sequencing	Shannon Index, observed species, Simpson & Chao1 indices, ↓.	PLS-DA & PCoA, significant differences.	1. Phylum: Bacteroidetes; 2. Class: Actinobacteria & Bacilli; 3. Order: Lactobacillales; 4. Family: Ruminococcaceae, Enterococcaceae & Lactobacillaceae; 5. Genus: *Bacteroides*, *Ruminococcus*, *Subdoligranulum.*	1. Phylum: Actinobacteria; 2. Class: Negativicutes & Bacteroidia; 3. Order: Bacteroidales & Selenomonadales; 4. Family: Lanchnospiraceae, Bacteroidaceae & Veillonellaceae; 5. Genus: *Lachnoclostridium.*
Vogt et al., 2017 [25]	USA	25 AD 25 Control	16S rRNA amplicon sequencing	ACE, Chao1, Shannon & Faith’s PD, ↓.	PCoA, significant differences.	1. Phylum: Bacteroidetes; 2. Genus: *Blautia*, *Bacteroides*, *Alistipes*, *Phascolarctobacterium*, *Bilophila*, *Gemella.*	1. Phylum: Firmicutes & Actinobacteria; 2. Genus: *Bifidobacterium*, *SMB53*, *Dialister*, *Clostridium*, *Turicibacter*, *Adlercreutzia*, *cc115.*

Note: MCI, mild cognitive impairment; AD, Alzheimer’s disease; ACE, abundance-based coverage estimator; PD, phylogenetic diversity; PCoA, principal coordinate analysis; CAP, constrained analysis of principal coordinates; NMDS, non–metric multidimensional scaling; PLS-DA, partial least square-discriminant analysis; PCA, principal components analysis.

In terms of the differentially abundant taxa, human cohort studies have also revealed associations between the abundance of specific gut bacteria and AD (Table). Most of these studies have identified differences at the phylum or genus level. For example, in one study conducted by Vogt et al. [25], increases in the abundance of the phylum Bacteroidetes and the genera *Blautia* and *Bacteroides* and decreases in the abundance of several taxa, such as Firmicutes and Actinobacteria at the phylum level and *Bifidobacterium*, *Clostridium*, and *Turicibacter* at the genus level, were observed in patients with AD. Importantly, these differentially enriched taxa were putatively linked to the biomarkers of AD pathology in cerebrospinal fluid, indicating a mechanistic link between alterations in the microbiota and neurological symptoms of AD. In another cross-sectional study conducted in Japan, the authors found that patients with dementia had a higher Firmicutes/Bacteroidetes ratio than healthy individuals and a slightly higher abundance of *Lactobacillus* and *Bifidobacterium* [27]. Instead of concentrating on a single gut microbiota stratification approach, a recently published Turkish cohort study [36] analyzed stool samples from 125 participants (27 patients with MCI, 47 patients with AD, and 51 healthy controls) and applied 4 machine learning analyses. They found that the abundances of *Prevotella* and *Bacteroides* were negatively correlated with AD, thereby contributing to neuroprotective effects against the progression of AD.

Previously published Chinese cohort studies have confirmed that patients with AD have definable alterations in their gut flora compared with healthy individuals (Table 1), with variations seen in subgroups of patients with MCI and AD [26, [28], [29], [30], [31], [32], [33], [34], [35], 37, 38]. One recent systematic review [39] of the gut microbiota in patients with AD presented conflicting evidence and concluded that *Proteobacteria*, *Bifidobacterium,* and *Phascolarctobacterium* showed significantly high abundance in patients with AD, whereas Firmicutes, Clostridiaceae, Lachnospiraceae, and Rikenellaceae showed significantly low abundance in patients with AD. Subsequently, a cohort study [30] of 56 participants (18 with AD, 20 with MCI, and 18 age-matched healthy controls) also found microbiota changes in those with AD and MCI. The abundance of 3 genera (*Bacteroides*, *Lachnospira,* and *Ruminiclostridium*) was reduced and one genus (*Prevotella*) was enriched in patients with AD, whereas only the genus *Lachnospira* showed significantly lower abundance in patients with MCI. However, a negative correlation between the abundance of *Prevotella* and cognitive function was observed in both patients with MCI and AD. Importantly, another study [28] of 171 people analyzed the clinical indicators and structure of stool microbiota and demonstrated that the clinical indicators of AD were positively correlated with the abundance of butyrate-producing bacteria, such as *Faecalibacterium*, but negatively correlated with the abundance of lactate- and propionate-producing bacteria, such as *Bifidobacterium* and *Akkermansia*, respectively.

There is evidence indicating that the essential functions of the gut microbiome may be strain-dependent, which imposes limitations on 16S RNA analysis because the typical sequencing depth is only at the genus level. Thus, more studies of causality are required using advanced shotgun metagenomics methods. Strikingly, in a prospective cohort study based on metagenomic analysis using the NextSeq 500 metagenomic analysis, Haran et al. [40] found that older individuals with AD have a lower abundance of key butyrate-producing species than healthy subjects. Moreover, using a machine learning method, the authors combined metagenomic data with clinical indices and found that the intestinal health of patients with AD may be affected by fecal microbiota through the P-glycoprotein pathway. This ground-breaking research filled a knowledge gap and provided a causal link between the microbiome and inflammation, which may underlie the pathogenesis of AD.

Although these human studies from different countries provide direct evidence supporting the contribution of the microbiome to AD, further longitudinal studies of large cohorts that explore the causal association between alterations of microbiota and AD are warranted.

## Mechanistic Insights into the Gut Microbiome, AD, and Dietary Components

Given the complex interactions and biological systems involved in gut–brain connections, multiple pathways may act together to modulate numerous aspects of the disease. In this section, we build on foundational observations to discuss how the microbiome affects and mediates key brain processes in AD, highlighting the complex intersection between various communication modalities.

### Neuronal signaling via the gut–brain axis

**FIGURE 1:**
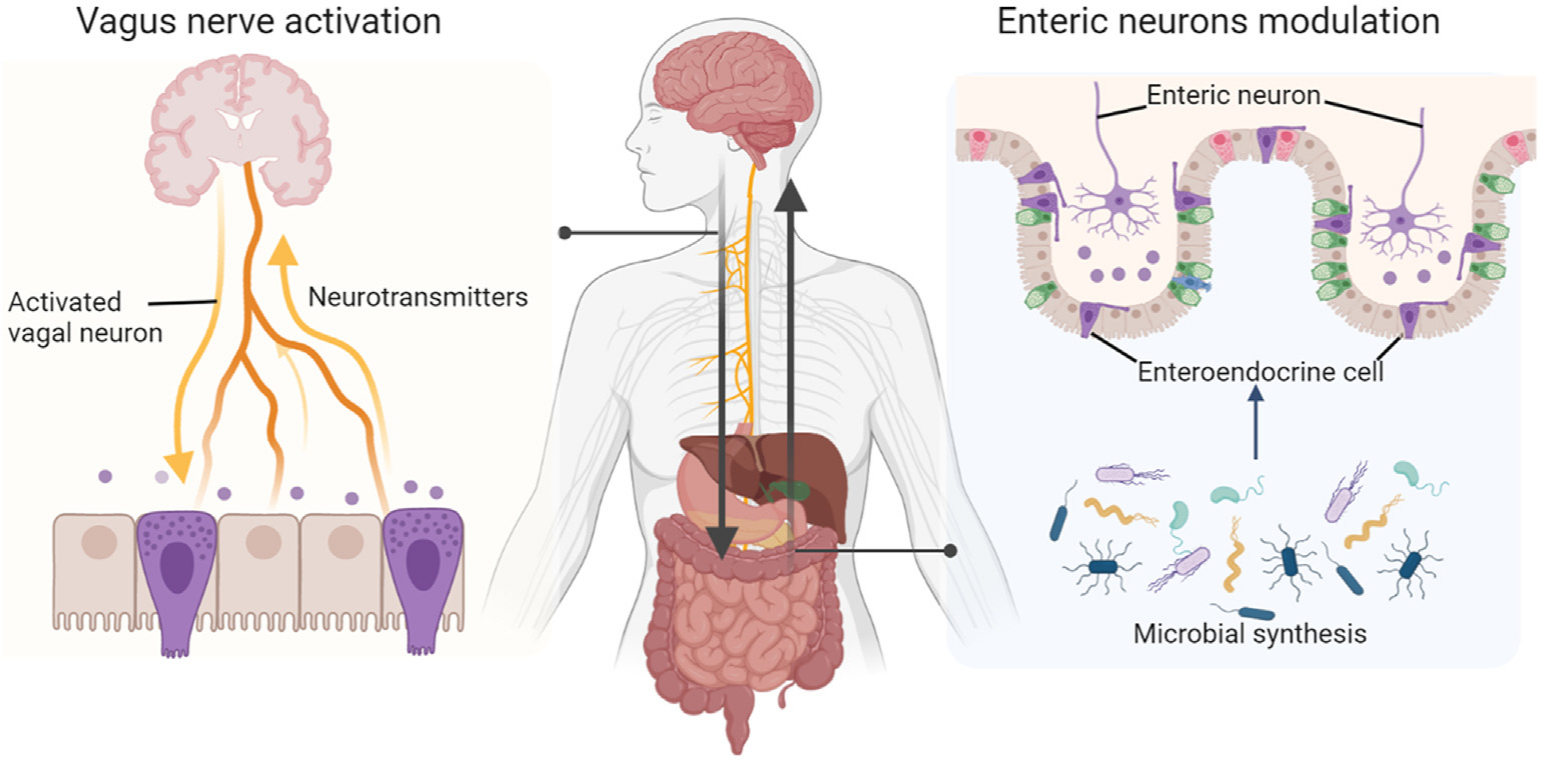
Neuronal signaling for gut–brain interactions. The neuronal pathways between the gut microbiota and the central nervous system (CNS) bidirectional communication involve the vagus nerve activation (left) and enteric neurons modulation in the enteric nervous system (right).

As a bidirectional mode of communication between the gut bacteria and the brain, the gut–brain axis is important for maintaining the homeostasis of every organ system within the body, including the gastrointestinal, microbial, and central nervous systems (CNS) [10, 41]. As described below and shown in Figure 1, the pathways of communication in these systems include neuronal pathways via the activation of the vagus nerve and interactions with the enteric nervous system (ENS).

#### Vagus nerve activation

The gut and brain are directly physically linked by the neuronal signaling pathway, mainly through the vagus nerve. The vagus nerve originates in the brainstem and innervates the gastrointestinal tract along the muscular layers, where it detects sensory signals and relays them to the CNS to affect behavior. This has been described in several animal studies [[42], [43], [44]].

For example, the administration of *Lactobacillus reuteri* has been shown to rescue social deficits in a vagus nerve-dependent manner in different autism spectrum disorder models [42]. Similarly, another study showed that oral *L. rhamnosus* JB-1 supplementation suppresses the expression of the gamma-aminobutyric acid (GABA) receptor in the hippocampus and amygdala as well as modulates behavioral dysfunction in mice with depression [43]. However, the neurochemical effects of *L. rhamnosus* JB-1 were ablated in vagotomized mice. These findings demonstrate the crucial effects of the vagus nerve on behavior and highlight the possibility of altering neurological activity through the activation of the vagus nerve. Interestingly, one cohort study reported that patients who underwent full truncal vagotomy exhibited a decreased risk for subsequent PD [44]. Despite the approval of using vagus nerve stimulation via surgical implantation in treatments for epilepsy and depression, the safety, tolerability, and efficacy of such treatments remain unclear. Although the microbiota-based stimulation of the vagus nerve to improve neurological activity is promising, details of the mechanisms need to be elucidated in animal models. In addition, the translation of the vagus nerve stimulation findings to humans remains a major challenge owing to the difficulties and complexities in investigating the vagus nerve in humans.

#### Enteric nervous system

The gut microbiome is also connected to the brain through the ENS, which is the largest component of the peripheral nervous system [45]. Using GF mice, a recent study found that *Bacteroides thetaiotaomicron* conventionalization restored the disordered ENS and increased the number of glial cells [46]. Similar findings were reported in another study in which the organization and properties of enteric neurons were altered in GF mice and modified by microbiota colonization [47]. These findings demonstrate that gut microbes regulate the development of ENS. Intriguingly, the microbiota can modulate sympathetic neurons through the certain gut–brain circuits and suppress gut–brain connections, as shown by recent evidence of microbiota depletion activating gut-extrinsic neurons [48].

Although ENS is a crucial modulator of enteric homeostasis and a regulator of gut barrier function, its central role in neurodegenerative diseases is less understood. One study examined the peripheral tissues of older individuals and observed a negative correlation between the appearance of pathological neurofibrillary tangles and phosphorylated tau expression in the periphery [49]. In addition, another review summarized preclinical and clinical studies and reported that the alterations in the microbiome composition can trigger Aβ accumulation [50]. However, owing to the limited availability of gastrointestinal tissues from patients with AD, no human studies have been conducted to determine the effects of ENS in patients with AD. Therefore, further studies are warranted on the importance of clinical gastrointestinal symptoms in patients with AD and the potential role of ENS in AD pathophysiology.

As the CNS and ENS are similar in structure and may be affected in parallel, diseases may originate in the ENS and involve the CNS as they progress. Therefore, further studies are required to determine whether modulating the neuronal pathways of the gut–brain axis can offer a novel approach for the prevention of AD.

### Immune-mediated signaling

**FIGURE 2:**
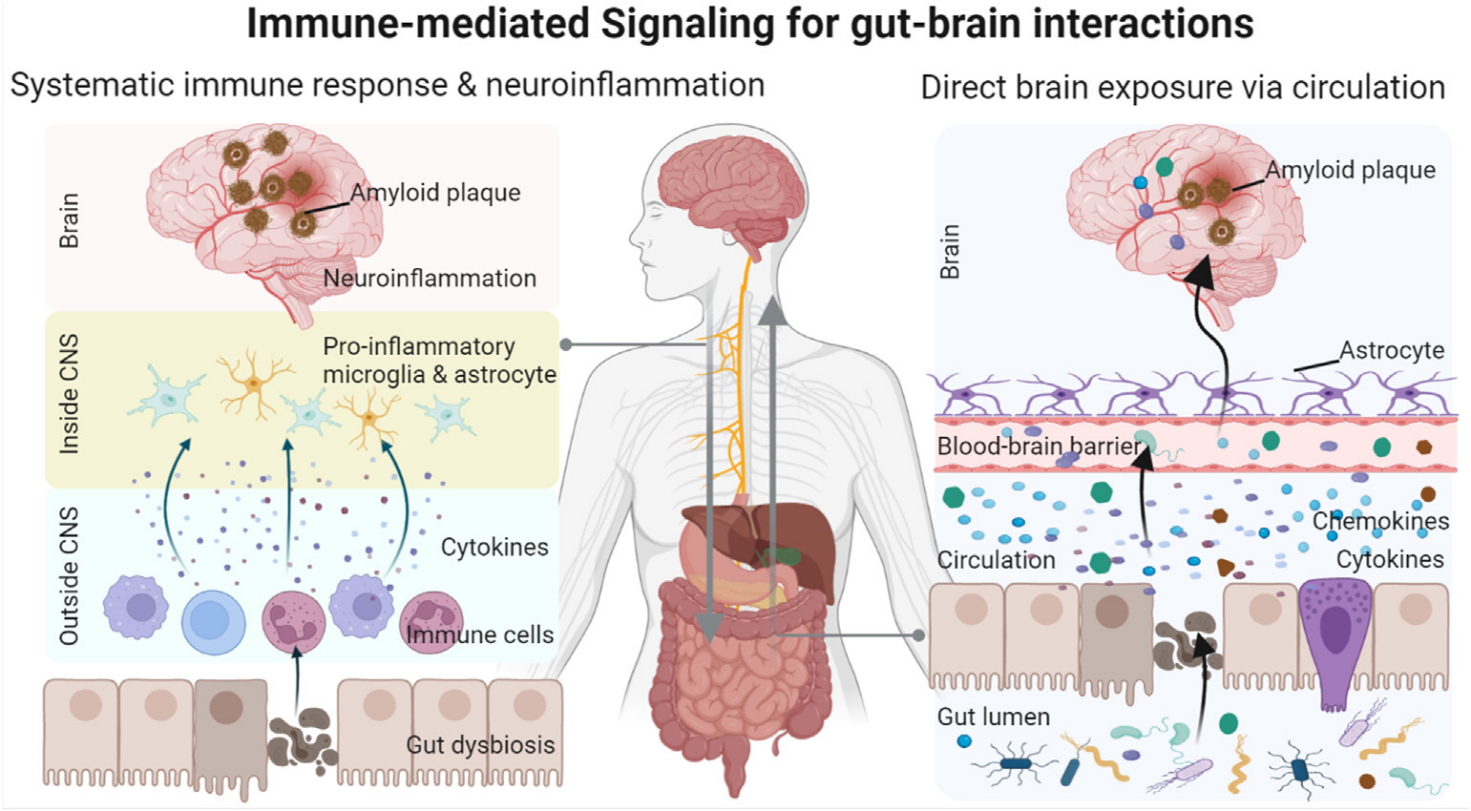
Immune-mediated signaling for gut–brain interactions. The gut microbiota is necessary for the maturation and activation of immune cells of the brain, and is a crucial factor to influencing the development and function of the neuroimmune system directly or indirectly. On the left, gut dysbiosis induces the production of cytokines by immune cells, which can lead to elevated neuroinflammation via directly entering the brain or by inducing an elevated systemic immune response. On the right, direct exposure of cytokines and chemokines to the brain via circulation may suppress neuroinflammation, which result in reduced amyloid plaques and improved cognition. CNS, central nervous system.

The gut microbiota contributes to the activation of immune cells in the brain and plays a critical role in the functioning of the neuroimmune system, both directly and indirectly [51]. Chronic exposure to inflammation, which is believed to be driven by increased intestinal permeability and gut bacteria dysbiosis, can affect various neurological disorders (Figure 2). Moreover, changes in systemic immunity result in increased neuroinflammation and altered immune signaling in neuropsychiatric diseases [52]. For instance, in a mouse model of maternal immune activation, the authors demonstrated gastrointestinal barrier defects and microbiota alterations, which were associated with behavioral and physiological abnormalities. However, *Bacteroides fragilis* supplementation reversed the core behavioral patterns and restored the microbial composition [53]. Using AD mouse models, alterations in the microbiota have been observed during the progression of AD. Furthermore, microbiota dysbiosis increases the concentrations of amino acids (AAs) in serum samples, which activates the differentiation and proliferation of pro-inflammatory T helper 1 cells. Further mechanistic studies have shown that brain-infiltrating T helper 1 immune cells are closely linked to the activation of M1 microglia, resulting in AD-related neuroinflammation. This finding highlights the role of gut dysbiosis-induced neuroinflammation in the progression of AD [54].

In addition to microglial or immune cell activation in the brain, the brain and intestinal microbiota communicate with the immune system through circulating cytokines (Figure 2) [55]. A large proportion of cytokines produced by brain-resident immune cells and microbiota-derived metabolites can cross the blood-brain barrier (BBB) and thus be transferred to the brain and affect brain function and host health. The integrity and permeability of the BBB are increased after exposure to infections and neurological diseases, thereby increasing the accessibility of the brain to cytokines and chemokines [56]. Importantly, there is evidence to show that age-induced peripheral and hippocampal immunity, along with the transplantation of microbiota from young mice, remodel the microbiome and promote the restoration of immune and brain functions; glutamine is believed to be a potential driver of this phenomenon. These findings likely represent the intimate connections between the microbiota, neuroimmune system, and outcomes in the brain. Future studies should aim to elucidate the direct mechanisms underlying these phenotypes.

### Microbial metabolites regulate AD

**FIGURE 3:**
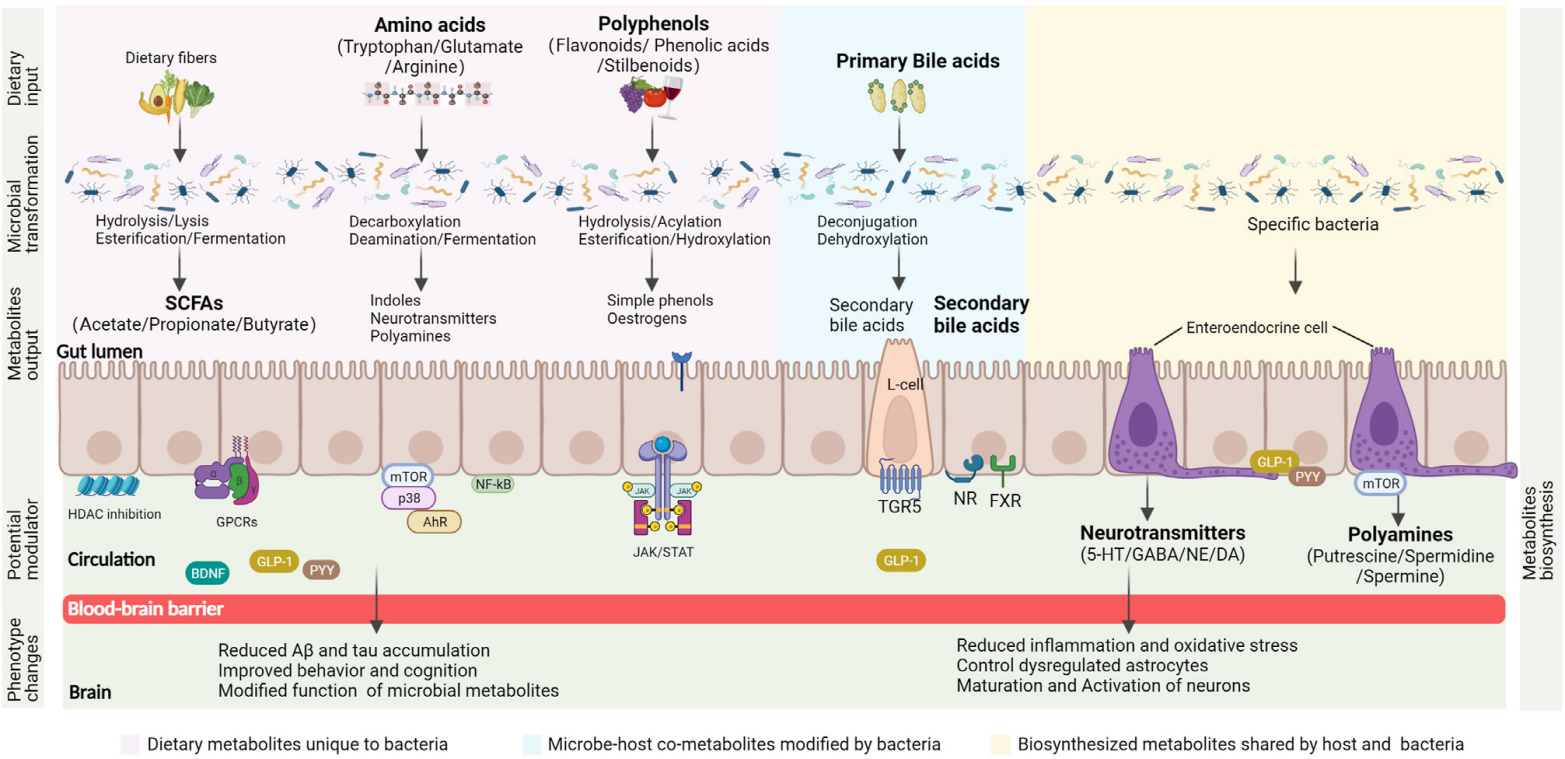
Mechanisms of microbial metabolites regulate AD through gut–brain communication. An overview of some major microbial metabolites act on epithelial, endocrinal and immune cells to affect gut microbiota composition, neuroinflammation, signaling transduction, cellular responses. After crossing the blood-brain barrier and reaching the circulation to tissue site or acting on circulating immune cells or afferent neuronal cells, these metabolites serve as messengers linking the gut to systems organs, which contributes to the function and formation of the host brain. These microbial metabolites can be categorized into 3 types: dietary metabolites unique to bacterial metabolism, including SCFAs, amino acids, and polyphenols; microbe–host co-metabolites modified by bacteria, such as bile acids; and biosynthesized metabolites shared by host and bacterial metabolism, such as neurotransmitters and polyamines. SCFAs, short-chain fatty acids; HDAC, histone deacetylases; GPCRs, G protein-coupled receptors; GLP-1, glucagon-like peptide 1; PYY, peptide YY; BDNF, brain-derived neurotrophic factor; mTOR, mammalian target of rapamycin; AhR, aryl hydrocarbon receptor; JAK, Janus kinase; STAT, signal transducers and activators of transcription; TGR5, Takeda G protein-coupled receptor 5; NR, nuclear receptor; FXR, Farnesoid X receptor; 5-HT, 5-hydroxytryptamine; GABA, γ-aminobutyric acid; NE, norepinephrine; DA, dopamine.

Emerging studies have revealed the signaling role of a series of metabolites in regulating neurological processes. These microbial metabolites can be broadly categorized into 3 types: diet-derived products, microbe–host co-metabolites, and metabolites shared by the host and bacterial metabolic pathways [57]. In this section, we used examples with the strongest evidence in each category to discuss the potential pathways by which microbial metabolites regulate brain function in AD (Figure 3).

#### Transformation of dietary metabolites

Dietary nutrients and components that are poorly absorbed by the host can still reach the colon and are metabolized by the gut microbiota into bioavailable and bioactive metabolites. The 3 classes of dietary metabolites with the most evidence for gut–brain interactions are SCFAs, AAs, and polyphenols.

#### SCFAs

SCFAs are the major end products of microbial fermentation of indigestible foods in the gut. Complex carbohydrates, especially dietary fibers, are usually considered the key substrates for SCFA production. The most abundant SCFAs in the gut are acetate, propionate, and butyrate, which constitute more than 95% of the SCFA content and are present in an approximate ratio of 3:1:1. Most SCFAs are produced in the caecum and proximal colon. After rapid absorption by colonocytes, SCFAs are used as energy substrates. Excess SCFAs that are not absorbed are transported into the portal-systemic circulation, thereby directly reaching other tissues.

A wealth of evidence demonstrates that SCFAs exert numerous beneficial effects on intestinal homeostasis and systemic metabolism. For instance, the concentration of SCFAs was significantly lower in stool samples from patients with irritable bowel syndrome with constipation than those from healthy controls, owing to the increased production of bacterial SCFAs [58]. As reported in a review article, in addition to their local effects on gut health, SCFAs can reach the brain, where they mediate gut–microbiota–brain interactions by binding to G-protein-coupled receptors [59]. Importantly, dietary supplementation with SCFA-producing bacteria or SCFAs markedly improves brain function. For example, in a mouse model of AD, butyrate administration at a concentration of 15 mg/kg.bw daily has been shown to prevent memory impairment and inhibit neuronal amyloid formation through the gut–brain axis [60]. Supplementation with the probiotic strain *Bifidobacterium breve* CCFM1025 has been shown to increase SCFA levels in faces, improve memory and behavior, and ameliorate neuroinflammation [61].

Moreover, SCFAs directly affect gene expression by inhibiting histone deacetylases (HDACs), which are key enzymes in neural processes. Evidence demonstrating that butyrate and propionate are potent inhibitors of HDACs has mostly come from animal studies [62, 63]. Butyrate treatment at a concentration of 1.2 g/kg.bw daily improved memory and reduced inflammation in a mouse model of AD via the inhibition of HDACs [63]. As patients with AD have shown increased expression levels of HDACs in the hippocampus compared with healthy individuals [64], the pharmacological use of SCFAs to inhibit HDACs should be widely explored. Furthermore, SCFAs can cross the BBB and reduce neuroinflammation.

Taking these findings together, SCFAs can be considered promising candidates for promoting brain function and attenuating inflammation in AD. In addition to dietary interventions, microbiota manipulation via the ingestion of probiotics and prebiotics may be an effective strategy to delay the progression of AD and improve AD-associated behaviors.

#### AAs

AAs are precursors for the biosynthesis of important neurochemicals and neurotransmitters. The 20 proteinogenic AAs are divided into 2 groups: essential and nonessential AAs. As they cannot be biosynthesized, the 9 essential AAs must be obtained from the diet or other external sources.

Evidence for the crucial effects of AAs in regulating neurodegenerative diseases, particularly AD, in animals and humans has been accumulating. In 5×FAD transgenic mice, significant increases in AAs have been observed in the blood and feces [54]. Subsequent human studies have shown that patients with AD have markedly increased concentrations of phenylalanine, isoleucine, and arginine in the blood compared with healthy controls [54]. It is likely that the gut microbiome affects the brain and is involved in neurological disorders; the microbiome might partly exert its effect by modulating the availability of AAs through 3 major mechanisms: *1*) the production of AAs by fermentation of dietary nutrients, *2*) the utilization of AAs for protein synthesis or as energy sources for metabolism, and *3*) the metabolism of AAs by deamination and decarboxylation [11].

The most extensively studied AAs that potentially affect brain function are tryptophan, glutamate, and arginine. Tryptophan is metabolized by the microbiota through 3 major metabolic pathways: 2 predominant host pathways (serotonin [5-hydroxytryptamine, 5-HT] and kynurenine) and 1 well-characterized microbial pathway (indole derivatives) [65]. Recently, a narrative review has summarized the role of 5-HT in the development of AD via regulating the microbiota–gut–brain axis [66]. In in vivo and in vitro studies, the alterations of kynurenine metabolites, especially kynurenic acid and quinolinic acid, showed potential relevance in the impairment of cognitive function in AD and other neuroinflammatory diseases [67, 68]. Additionally, quantification of tryptophan metabolites in the urine and serum samples of individuals clinically diagnosed with AD revealed that significantly higher kynurenine levels and lower 5-HT levels may result in the systemic inflammation and modulation of the kynurenine and 5-HT pathways could help improve tryptophan bioavailability in patients with AD [69].

Although they are highly selective, many tryptophan metabolites, which have notable effects on the metabolism of neurotransmitters, cross the BBB and thus reach the brain. Arginine, a polyamine precursor, has been linked to neurodegeneration. A previous study found that arginine levels increase with age [24]. In contrast, another study showed that supplementation with the probiotic LKM512, in combination with arginine, had possible neuroprotective effects on the brain [70]. Consistently, a more recent study showed that both arginine supplementation (at a dose of 0.4 mg/g.bw/d) and *Bifidobacterium longum* strain administration significantly reversed age-related behavioral damage [71]. However, gut-derived 5-HT cannot cross the BBB. As an intermediate in the synthesis of 5-HT, periphery 5-hydroxytryptophan (5-HTP) can cross the BBB and be converted through a chain of reactions into 5-HT in the brain [66, 72].

Given their multifaceted and potentially detrimental effects, the regulation of brain function in AD by AAs is a complex phenomenon. Moreover, it is unclear whether AAs are causative factors or merely biomarkers of AD modulation. Therefore, further exploration of the mechanisms by which the bacterial transformation of AAs affects behavior and cognition is warranted.

#### Polyphenols

Polyphenols, including flavonoids, phenolic acids, and stilbenoids, are plant-derived metabolites that are essential for human health [73]. There are thousands of polyphenols, most of which are abundant in fruits and vegetables such as grape pomace, apples, berries, oranges, pomegranates, and tomatoes. Polyphenols are also present in coffee, tea, wine, and olive oil. However, most dietary polyphenols are poorly absorbed by the stomach and small intestine and are transported to the colon to be metabolized into bioavailable and bioactive metabolites by the gut microbiota [74]. After hydroxylation, these microbial metabolites are secreted into the bloodstream and transported to the peripheral tissues via systemic circulation to exert their biological effects [75].

Accumulating research has begun to demonstrate the beneficial effect of polyphenol treatment in ameliorating cognitive defects in AD and PD mouse models [[76], [77], [78], [79]]. Dietary supplementation with parent polyphenols has been shown to alter phenolic metabolite levels in the brains of different mouse models of AD [80]. Specifically, grape seed extract is a well-studied source of dietary polyphenols that was shown to attenuate cognitive impairment by preventing amyloid and tau aggregation in mouse models of AD [81]. Moreover, the gut microbiota promotes the beneficial effects of polyphenols in neurological diseases. For instance, in rats with AD, grape seed polyphenolic extract administration significantly increased the levels of bacterial metabolites in the brain [79]. Continuing this work, a recent study of a mouse model of PD showed that treatment with plant-derived epigallocatechin gallate effectively prevented amyloid aggregation and motor impairment and that these effects were promoted by gut bacteria [82]. Another double-blind, randomized controlled trial (RCT) study reported that epigallocatechin-3-gallate combined with cognitive training significantly improved cognition and behavior in young adults with Down syndrome [83]. These findings indicate that polyphenols confer neuroprotective effects against the development of AD by decreasing inflammation and altering metabolite concentrations.

Overall, polyphenols potentially alleviate AD and age-related pathology through microbiota modulation and dietary intervention. Despite the evidence for complex polyphenol–microbiota interactions, the direct effects of selective polyphenols on brain function and behavior and the associated pathways remain to be conclusively demonstrated.

#### Modification of microbe–host co-metabolites

In addition to the transformation of dietary metabolites, the modification of microbe–host co-metabolites, particularly bile acids, by the gut microbiota and their subsequent effects on the brain were summarized in a previous review [84].

#### Bile acid

Bile acids are a diverse class of signaling molecules that are synthesized from cholesterol in the liver (primary bile acids). Once secreted into the intestine and colon, they are further modified by gut bacteria (secondary bile acids) through dihydroxylation and deconjugation [85]. In the jejunum and colon, conjugated bile acids are reabsorbed through the enterohepatic circulation and can cross the BBB through active transport, whereas unconjugated bile acids are reabsorbed into intestinal enterocytes by passive diffusion [86]. Bile acids are recognized as the endogenous ligands of nuclear receptors [87].

Circulating bile acids can affect neuronal activity in the brain [88]. There is evidence supporting the notion that bile acids are neuroactive molecules and that they can directly bind to nuclear receptors in the brain or activate gut receptors to release signals, resulting in physiological effects [89, 90]. The protective effects of bile acids in several neurodegenerative and neurological disorders have been discussed in a recently published review [91]. In fact, all the secondary bile acids modified by the gut microbiota have been detected in the brain samples of patients with AD [92]. Importantly, the cognitive decline of patients with AD was found to be closely associated with increased levels of secondary bile acids in a cohort study of 1464 patients including those with MCI and AD [93]. Decreased concentrations of bile acid precursors have also been identified in the cerebrospinal fluid of patients with AD using liquid chromatography-mass spectrometry [94].

Moreover, early studies describing how microbiota-derived bile acids affect brain phenotypes, coupled with the identification of the microbiome and metabolome, have laid the foundation for pioneering research on gut microbes as the key regulators of bile acid metabolism [95]. In both mouse model and human studies, changes in bacteria-derived bile acids have been observed in AD [92, 93]. In addition, a separate study revealed that the gut microbiota increased the concentration of deoxycholic acid, resulting in the generation of neurotransmitters in gut enterochromaffin cells; this suggests a direct mechanistic link between the microbiota, bile acids, and neurological function [96]. In summary, these findings provide insights into the possible roles of bile acids in regulating AD, either directly or indirectly. Considering that bile acids may have either beneficial or detrimental effects, further investigation of the cause-and-effect relationship is warranted.

#### Biosynthesis of metabolites shared by the host

In addition to dietary metabolites and bile acids, there may be many additional host-associated microbes that affect neuronal activity and behavior via neurotransmitters and polyamines. Knowledge of these metabolite disturbances in AD has led to new treatments with microbiota-modulation potential, which have now come to testing in clinical trials.

#### Neurotransmitter

Neurotransmitters are a group of endogenous chemicals that are released by neurons. When released, they trigger nerve impulses to stimulate neighboring neurons or muscles, allowing chemical signals to be transmitted throughout the nervous system. Based on their chemical and molecular properties, neurotransmitters are divided into 4 major types: AAs, monoamines, peptides, and purines.

Neurotransmitters are essential for neurological functions and behaviors. The 2 most extensively studied neurotransmitters are 5-HT and GABA. Few studies have demonstrated the role of neurotransmitters in mediating the effects of gut bacteria. Reduced biosynthesis of 5-HT has been observed in both GF and antibiotic-treated mice; treatment with spore-forming bacteria has been shown to rescue this condition [96]. More recently, a human study demonstrated the presence of GABA-modulating bacteria in the microbiota [97]. Although these initial findings suggest that host-associated bacteria regulate neurotransmitters, little is known about the pathways underlying the microbial response to neurotransmitters. Moreover, abnormalities in neurotransmitters have been closely linked to various diseases, particularly neuropsychiatric and neurodegenerative disorders. For instance, decreased levels and activity of norepinephrine, GABA, and 5-HT are known to cause depression [98].

Importantly, the microbiome-targeted modulation of neurotransmitters is believed to protect the host from brain pathologies. As a primary neurotransmitter of the parasympathetic nervous system, acetylcholine is essential for processing memory and learning. The acetylcholinesterase inhibitor, donepezil, effectively alleviates the cognitive and behavioral symptoms in patients with AD [99]. GABA is known to dampen neuronal activity [100]. A more recent study using untargeted metabolomics showed that *B. breve* CCFM1025 supplementation alters glutamate concentrations in both the serum and hippocampus of a mouse model of AD [101]. In addition, in patients with depression, supplementation with *B. breve* CCFM1025 was shown to attenuate depressive symptoms by regulating 5-HT and the gut microbiota composition [102].

Overall, modulating the concentrations of neurotransmitters via microbiota–brain neuronal signaling is a potent approach to regulating memory and behavior. Despite evidence that neurotransmitter levels regulated by certain host-associated bacteria can affect brain function, the extent to which microbial modulation directly affects neuronal activity and behavior requires further investigation.

#### Polyamines

Polyamines are polycationic bioactive molecules that can be metabolized by the intestinal microbiota. The most well-characterized polyamines with modulatory effects on human health are putrescine, spermidine, and spermine [103].

Agmatine is a polyamine precursor and ligand of imidazoline receptors in the brain [104]. Supplementation with agminate or agminate-producing *Escherichia coli* effectively improved host health and longevity through drug–nutrient–microbiome interactions in mice [105]. Moreover, emerging evidence highlights the crucial role of polyamines in regulating brain function in AD, such as memory formation, synaptic plasticity, and behavior. In mouse models of AD, polyamine levels increase in the hippocampus, which is accompanied by increases in the activity and expression levels of arginase [106]. Similar findings have been observed in the cortices of patients with AD, which have lower levels of arginine than in the cortices of control individuals [107].

Moreover, polyamines may improve brain function through microbiota manipulation, such as by probiotic supplementation. One study found that the administration of the *Bifidobacterium animalis subsp. lactis* LKM512 and arginine increased colonic putrescine and serum polyamine levels as well as ameliorated inflammation and memory impairment [70]. Another study showed that *Bifidobacterium animalis subsp. lactis* LKM512 supplementation effectively ameliorated age-related colonic inflammation by upregulating polyamine production in the intestinal lumen [108]. Taken together, these findings suggest that polyamines are potent regulators of AD through dietary intervention and microbiota manipulation. Therefore, given their multifaceted effects on several different, but interconnected, pathways and their interactions with whole-body systems, the regulation of behavior and memory by polyamines in AD may be complex [109].

As we do not have a clear understanding of the extent to which the microbiota directly or indirectly affects brain activity and behavior, it is difficult to separate the different signaling pathways. As such, all communication pathways comprising gut microbiota–brain connections are considered co-opted by the microbiome to affect the brain and to be intertwined with other pathways.

## Microbiome-based Dietary Interventions to Delay the Progression of AD

**FIGURE 4:**
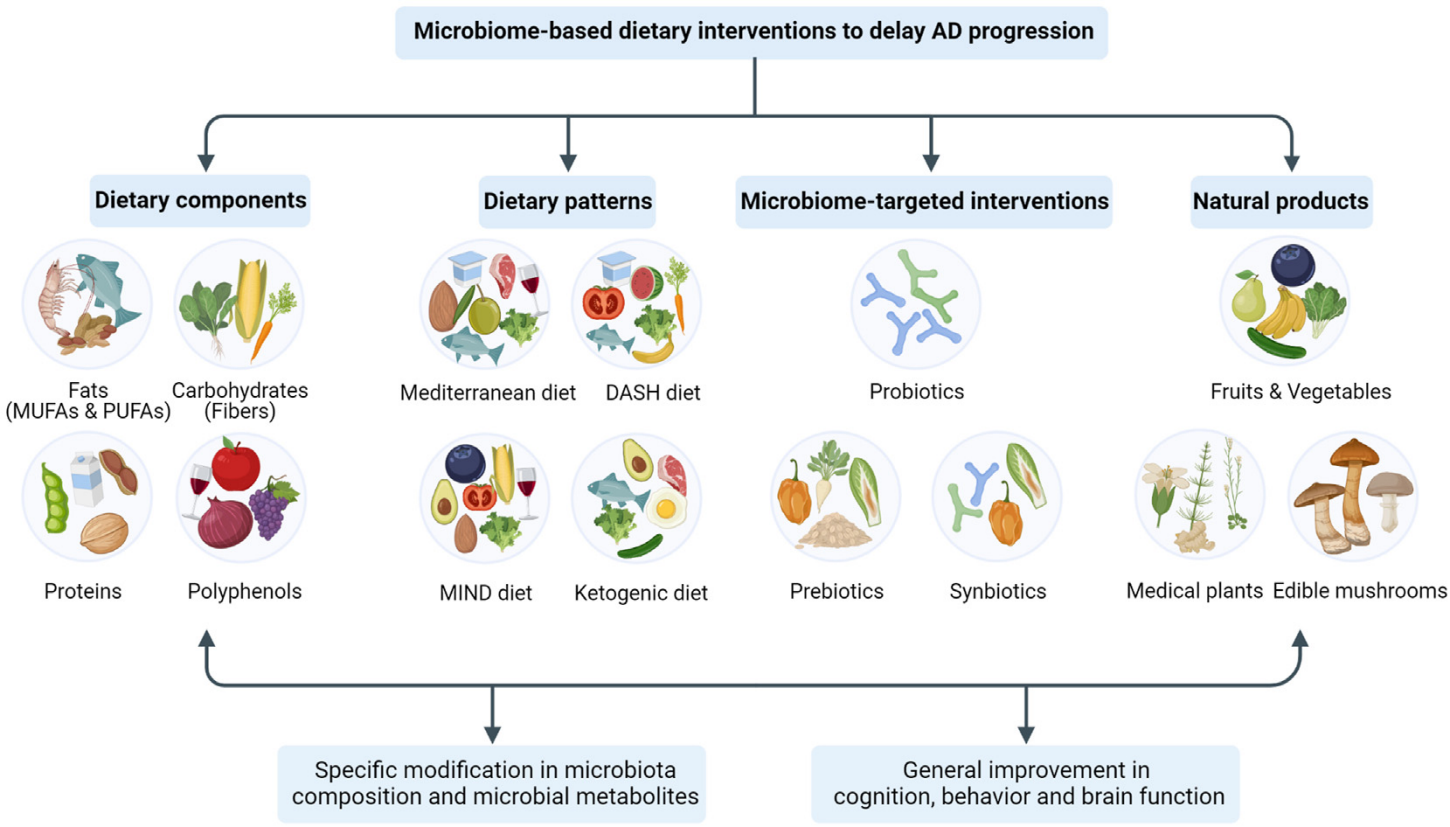
Microbiome-based dietary interventions to delay the progress of AD. AD, Alzheimer’s disease; MUFAs, monounsaturated fatty acids; PUFAs, polyunsaturated fatty acids; DASH, dietary approaches to stop hypertension; MIND, Mediterranean–DASH intervention for neurodegenerative delay.

A greater understanding of the metabolic and nutrient requirements of beneficial members of the gut microbiota creates the possibility of supporting these species through targeted dietary supplementation. In fact, alterations in dietary components or patterns, microbiome-targeted intervention, and natural product supplementation effectively alter the microbiota composition and consequently, alleviate AD-related pathology (Figure 4).

### Dietary components

Dietary components such as fats, carbohydrates, proteins, vitamins, and polyphenols, which are not absorbed completely by the small intestine, can be transported to the colon and then processed by the gut microbiota [110]. Major changes in individual dietary components can significantly alter the microbiota composition and promote metabolite production. As gut dysbiosis, inflammation, and cognitive impairment are features of AD, the modulation of the microbiota through specific dietary components offers an effective strategy to prevent AD.

#### Fats

Dietary fats are either saturated or unsaturated, each of which can have different effects on the gut microbiome and brain health. The functions of numerous beneficial bacteria are altered based on the consumption of certain types of fats, such as FFAs, MUFAs, and PUFAs [111]. Animal studies have shown that the consumption of fish-oil, which is rich in omega-3 PUFAs, enriches beneficial microbes such as *Bifidobacterium*, *Lactobacillus,* and *Akkermansia muciniphila*, which may decrease metabolic inflammation [112]. Similarly, a recent human study found that omega-3 PUFA supplementation increases the prevalence of SCFA-producing bacteria at the genus level, such as *Bifidobacterium*, *Roseburia*, and *Lactobacillus* [113]. Moreover, findings from interventional studies have indicated the protective role of PUFAs and MUFAs against AD-associated cognitive decline [114]. For example, docosahexaenoic acid supplementation has been shown to attenuate Aβ accumulation and neuroinflammation in 5×FAD mice, whereas omega-3 PUFA administration has been shown to markedly alleviate Aβ-induced mitochondrial pathology [115]. In a series of cohort studies, dietary intervention with seafood or omega-3 PUFAs was shown to ameliorate cognitive decline in individuals with MCI and AD [116, 117]. However, the results from an epidemiological study indicated that the supplementation of omega-3 fatty acids by those who carry APOEε4 appears to differ by age, sex, and disease stage [118, 119]. Considering that the daily dosage and duration of omega-3 PUFA consumption differ across studies, the beneficial effects and the extent to which cognitive defects are alleviated may also differ across these studies.

Taken together, these findings indicate that a diet containing specific fats, especially omega-3 PUFAs, can directly affect the microbiota composition and regulate cognition and brain function in AD.

#### Carbohydrates (fibers)

Carbohydrates can be broadly divided into simple sugars and complex carbohydrates. High levels of sugar decrease the diversity of gut microbes and increase the Firmicutes/Bacteroidetes ratio, both of which are closely linked to cognitive function [120]. Studies in animals and humans have demonstrated the relationship between a high-sugar diet and AD; the long-term consumption of such a diet can induce neuroinflammation and promote learning and memory deficits [121, 122]. In contrast, in animal studies, the intake of dietary fiber consisting of complex polysaccharides has been linked to protective effects on brain function and memory through fermentation by the gut microbiota and the inhibition of neuroinflammation [123]. Evidence from human studies involving dietary supplementation with different types of fiber has also supported the notion that the intake of dietary fiber has beneficial effects on brain health via different mechanisms, depending on the type of fiber [124]. This may partially be due to the fact that a plant-based diet increases the abundance of probiotic bacteria (*Bifidobacterium*, *Lactobacillus,* and *Roseburia*), which further metabolizes fiber and complex polysaccharides into SCFAs, which are then transported to the brain via the systemic circulation to affect behavior and cognition [125].

#### Proteins

Unabsorbed dietary protein reaches the colon, where it is fermented by proteolytic bacteria into beneficial end products that affect host function and microbiota composition [126]. However, as an essential nutrient, the impact of dietary protein on microbial metabolism can differ between individuals depending on the content, protein type or source, and amount of protein consumed [127]. Similarly, in patients with AD, the consumption of a large amount of animal protein has been shown to increase cognitive decline and have detrimental effects on brain health [128]. However, the consumption of plant proteins has been shown to decrease the prevalence of AD and exert protective effects on cognitive function [129]. In addition, the protein source is also considered a critical determinant of microbial metabolism, which may be detrimental for brain health. For instance, a diet rich in animal-derived protein has been shown to increase the production of neurotoxic end products (e.g., hydrogen sulfide and ammonia), indicating that animal protein-derived metabolites can cause inflammation [130]. In contrast, the consumption of plant-derived protein was shown to decrease the prevalence of pathogenic taxa such as *Bacteroides fragilis* and *Clostridium perfringens* and increased the prevalence of beneficial taxa such as *Bifidobacterium* and *Lactobacillus* [131]. However, it is worth noting that plant-based foods such as whey and soy are abundant in oligosaccharides and fibers. Thus, it is challenging to determine the direct effect of plant proteins on brain function.

#### Vitamins

Vitamins are essential micronutrients with potential antioxidant and neuroprotective properties. They are also known to affect the composition and diversity of the gut microbiota [132]. Humans require 13 vitamins, of which 4 are classified as fat-soluble (vitamins A, D, E and K) and 9 as water-soluble (8 B vitamins and vitamin C). Indeed, fat-soluble vitamins, vitamins A and D in particular, have gained attention for their effects on the immune system when absorbed in the intestinal tract and secreted into the bloodstream [133]. A recent cross-sectional analysis of 567 older individuals showed that butyrate-producing bacteria are more abundant in people with high levels of vitamin D [134]. Vitamin E (mainly tocopherols) levels have been linked with MCI and AD risks [135, 136]. Additionally, a large cohort study of aging people reported that vitamin B1 and B6 metabolism were closely associated with cognition and brain structure and function [137]. Despite these evidence, few studies have evaluated the effects of vitamins on the gut microbiome, and the causal role of vitamins in modulating the microbiota remains poorly understood.

Moreover, efforts to explore the functional roles of dietary vitamins in regulating brain function in neurological diseases have paved the way for clinical interventions [138]. In lipopolysaccharide-treated aged rats, dietary supplementation with retinoic acid, a nutritional metabolite of vitamin A, was shown to suppress the production of nitric oxide and downregulate nitric oxide (NO) synthase levels in the cortex and hippocampus, which in turn affects cognition and brain function [139]. In addition, vitamin D supplementation for 3–6 months has been shown to improve cognition and memory in individuals with AD or dementia [140]. However, a recently published research article reported contrary results that vitamin D supplementation may worsen the progression of AD, using an APP/PS1 mice model and human cohort studies [141]. The results of one observational and Mendelian randomization study showed that low vitamin D status was associated with the risks of dementia [142]. However, it should be noted that a large-scale RCT of 25,871 older participants failed to show a link between decreased systematic inflammation and vitamin D consumption [143]. Owing to differences in the dosage and duration of treatment in different studies, the observed neuroprotective effects may differ. Thus, additional well-designed prospective studies are warranted [144]. Furthermore, the production of vitamins by the microbiota and their requirement for brain development must be clarified with future studies that must identify possible signaling pathways through which vitamins affect brain function.

#### Polyphenols

As previously mentioned, most polyphenols have low bioavailability and unabsorbed dietary polyphenols can alter the function of the gut microbiota. Findings from several studies have demonstrated the effect of polyphenol-rich foods on gut microbes. For example, blueberry supplementation has been shown to markedly enrich probiotic bacteria such as *Bifidobacterium* and *Lactobacillus*, which are closely linked with decreases in systemic inflammation in rats [145]. Similar alterations of the gut microbiota and its metabolites have also been recorded after dietary supplementation with other polyphenol-rich foods, including, but not limited to, grape seed extract, green tea, pomegranates, coffee, red wine, and phenolic compounds such as curcumin, resveratrol, epigallocatechin gallate, anthocyanins, allicin, and flavonoids [15]. Mechanistic studies have shown that dietary polyphenols exert neuroprotective effects on brain function by modulating synaptic plasticity and inhibiting peripheral inflammation [146]. Dietary supplementation with coconut oil, which contains flavonoid compounds with antioxidant properties, has been shown to reverse age-related neuronal disorders and ameliorate neuroinflammation and AD-induced cognitive decline [147]. Additionally, the consumption of grape seed extract attenuated cognitive impairment by preventing amyloid and tau aggregation in mouse models of AD. Resveratrol, which is abundant in grapes, soy, and nuts, has been shown to act as a signaling molecule via the gut–microbiota–brain axis to upregulate brain-derived neurotrophic factor expression, reduce the Aβ burden and improve brain function by modulating the microbiota and neurotransmitters in mouse models of AD [148].

Overall, dietary polyphenols change the function and composition of the microbiota and its metabolites, thereby conferring beneficial effects on the brain and behavior via gut–brain interactions.

### Dietary patterns

In addition to the associations between certain dietary factors (such as individual nutrients or phytochemicals) and the risk of AD, the examination of the diet as a whole and dietary patterns may help identify greater overall effects on the intestinal microbiota and brain function [149]. As individual dietary patterns contain multiple types of food in different combinations and frequencies, efforts to determine its influence on host health and disease states are necessary, but challenging [150]. The 4 classes of dietary patterns with the most evidence for AD regulation are the Mediterranean diet, dietary approaches to stop hypertension (DASH), the Mediterranean–DASH intervention for neurodegenerative delay (MIND), and the ketogenic diet [151].

#### Mediterranean diet

The Mediterranean diet consists of a high level of consumption of fresh fruits, vegetables, fish, nuts, extra-virgin olive oil, and whole grains and a moderate level of consumption of red meat, dairy products, and wine. With a mixture of omega-3 PUFAs, vegetable protein, fermentable carbohydrates, and bioactive compounds, the Mediterranean diet favors the growth of saccharolytic microbial species and promotes a beneficial metabolite profile [152].

There has been an increase in evidence for the important role of the Mediterranean diet in preventing cognitive decline and reducing the risk of AD [[153], [154], [155]]. A meta-analysis of 34,168 participants concluded that a high level of adherence to the Mediterranean diet is associated with 17% and 40% reduced risk of MCI and AD, respectively [156]. Similarly, another meta-analysis of 15 dietary cohort studies reported that the Mediterranean diet significantly improved the cognition of older adults [157]. Although the mechanisms by which the Mediterranean diet exerts neuroprotective effects remain poorly understood, several studies have proposed potential pathways. Notably, the Mediterranean diet has been shown to promote the growth of fiber-degrading and SCFA-producing bacteria such as *Prevotella*, *Bifidobacteria*, *Eubacterium eligens*, and *Bacteroides* and restore the abundance of pro-inflammatory bacteria such as *Ruminococcus gnavus*, resulting in a high concentration of beneficial SCFAs and low concentration of toxic metabolites [[158], [159], [160]]. Together with bioactive compounds and phytochemical extract supplementation, these metabolites can be transported from the gut to the bloodstream and then to the brain by crossing the BBB. In the brain, they decrease Aβ and tau accumulation and enhance synaptic plasticity during the development of AD [161]. Overall, the Mediterranean diet is a potent candidate for improving cognition through the manipulation of the microbiota and its metabolites.

#### The DASH diet

The DASH diet is generally recommended to prevent hypertension. It differs slightly from the Mediterranean diet in that it does not involve the consumption of fat or alcohol and restricts the consumption of sodium and sweets. Several studies have reported an association between the DASH diet and cognition in individuals with MCI or AD [162, 163]. For example, a large cohort study of 16,144 older women reported that long-term adherence to the DASH diet is linked to improved cognition [162]. Another prospective cohort study also supported the findings of the consumption of the DASH diet slowing cognitive decline in individuals aged >65 y [163]. A meta-analysis confirmed the association between the DASH diet and cognitive decline, although the neuroprotective effects were less consistent [13]. However, no significant association with cognitive decline was observed for the DASH diet in a longitudinal cohort study [164]. The combination of the DASH diet and exercise is known to effectively suppress cognitive impairment, although the diet alone has no significant protective effects, pointing to a novel targeting strategy by which to prevent cognitive impairment through lifestyle interventions [165].

#### The MIND diet

The MIND diet is a combination of the Mediterranean and DASH diets. It is characterized by a high content of plant-based food and limited intake of animal products and foods with high saturated fat content. The MIND diet specifically emphasizes the consumption of berries and green leafy vegetables. One recently published review summarized the clinical trials investigating the beneficial impacts of the MIND diet on cognition in older people [151]. Early in 2015, Morris et al. [166] conducted a longitudinal study of 960 participants and found that consumption of the MIND diet was positively linked to slower cognitive decline. Moreover, a cross-sectional study in the United States reported a dose-response effect of the MIND diet on cognition [167]. An Australian longitudinal cohort study of individuals with MCI and AD demonstrated that the MIND diet, but not the Mediterranean diet, markedly prevented cognitive impairment after 12 y of follow-up [168]. Therefore, the MIND diet appears to be a promising intervention for the regulation of AD-related cognitive impairment.

#### Ketogenic diet

The ketogenic diet involves the consumption of foods low in carbohydrates, but high in fat and protein, to induce a state of ketosis. Data from both animal models and human studies provide some support for the link between the ketogenic diet and prevention of AD [169]. A review of 11 human studies concluded that ketogenic diet supplementation improves memory, executive function, and global cognition [169]. A small-scale clinical trial of 20 Japanese individuals showed that supplementation with a medium-chain triglyceride-based ketogenic diet for 12 wk significantly improved the immediate and delayed logical memory of patients with mild-to-moderate AD [170]. Another double-blinded RCT showed that ketogenic medium-chain triglycerides could improve cognition in MCI individuals [171]. A dose of 30 g/d of ketogenic medium chain triglyceride (kMCT) taken for 6 mo bypasses a significant part of the brain glucose deficit and improves several cognitive outcomes in MCI. More recently, Chu et al. [13] summarized human studies evaluating the effects of the ketogenic diet on MCI or AD and found that 14 of 15 studies reported significant improvements in cognitive function. However, it is important to note that prolonged fasting in older people may lead to the production of toxic methylglyoxal levels and ketoacidosis [172].

Only a few studies have demonstrated the essential role of the microbiome and its metabolites in modulating brain function in individuals on a ketogenic diet. For instance, in 2 different mouse models of seizure, the intestinal microbiota were altered by a ketogenic diet and required for the anti-seizure effects of the diet to manifest. Further investigation using microbiota transplantation and metabolomic analysis revealed an increase in the abundance of ketogenic diet-associated bacteria (*Akkermansia muciniphila* and *Parabacteroides* spp.) and in hippocampal GABA and glutamate levels [173]. A double-blinded RCT study performed in individuals with MCI revealed that a modified Mediterranean-Ketogenic diet can modulate the gut microbiome and metabolites in association with improved AD biomarkers in cerebrospinal fluid [174]. However, case reports have revealed adverse events in people consuming a ketogenic diet who show an increased risk of intestinal diseases such as constipation and reduced appetite. Taking these findings together, direct manipulation of the microbiota and metabolite production through the ketogenic diet offers another effective intervention for the prevention of cognitive decline in patients with AD.

### Microbiome-targeted interventions

Owing to our increased knowledge of the influence of gut microbiota on neurological diseases, there is emerging interest in testing microbiome-directed interventions in humans with impaired brain function and a disrupted gut microbiome. Over the past few decades, studies exploring the approaches by which to modulate the microbiome in patients with AD have focused mainly on probiotics, prebiotics, and synbiotics.

#### Probiotics

Probiotics are defined as “live microorganisms that when administered in adequate amounts, confer a health benefit on the host” [175]. Despite evidence to show that probiotic intervention improves cognitive impairment, studies investigating the use of probiotic supplements to prevent the progression of AD have yielded inconsistent results [[176], [177], [178]]. For instance, one multi-center, double-blind RCT conducted in Korea showed that *Lactiplantibacillus plantarum* C29 (DW2009) administration for 12 wk significantly enhanced cognitive function in individuals with MCI [179], whereas another study conducted in Japan failed to confirm these benefits [180]. These conflicting results were analyzed in several systematic reviews and meta-analyses, which demonstrated that probiotic supplementation improves cognitive function, especially in individuals with MCI [177, 181]. Moreover, only one RCT conducted by Hwang et al. [179] reported an alteration in the microbiota composition, but no significant alterations were observed in the abundance of *Bifidobacterium* spp. or *Clostridium* spp. after probiotic intervention. Despite the potential to promote cognition, not all probiotics have the same psychobiotic effect, and their beneficial effects may be strain-specific. Probiotic strains can have different effects on patients with different disease severities, especially those in whom the gut environment is disrupted to varying degrees by disease-related factors. Moreover, the dose and duration of probiotic consumption are 2 of the most important considerations for interventional studies. Thus, taking all these points into account can guide future probiotic intervention-based studies.

#### Prebiotics

A prebiotic is “a substrate that is selectively utilized by host microorganisms conferring a health benefit” [182]. The most extensively studied prebiotics are inulin, fructooligosaccharides, and galactooligosaccharides which can increase the prevalence of probiotic bacteria (*Lactobacillus* and/or *Bifidobacterium* spp.) and thus impart health benefits [182]. Multiple animal studies have demonstrated the protective effects of prebiotic administration on Aβ deposition, synaptic plasticity, and neuroinflammation markers, as well as on alterations in behavior and the microbiome and its metabolites in different animal models. For instance, in APP/PS1 transgenic mice, the administration of fructooligosaccharides alleviates Aβ accumulation and ameliorates cognitive deficits and neurodegeneration by modulating the gut microbiota [183, 184]. In addition, dietary inulin intervention increases the abundance of beneficial microbiota and promotes the production of SCFAs and tryptophan-derived metabolites, indicating its potential to prevent AD [185]. More recently, using the 5×FAD transgenic mouse model of AD, 8 wk of mannan oligosaccharide treatment was shown to markedly improve memory and cognitive function, which was functionally linked to restored microbiota composition and promoted SCFA production [186]. Moreover, supplementation with other prebiotics such as xylooligosaccharides [187], β-glucan [188], and oligosaccharides from *Morinda officinalis* [189] ameliorated AD-related cognitive impairment and neuroinflammation via microbiota modulation. However, no clinical research has been published on the effect of selective prebiotics in patients with MCI or AD. This may be because these prebiotics are usually added to a defined diet rather than administered themselves, in clinical trials.

#### Synbiotics

A synbiotic is defined as “a mixture comprising live microorganisms and substrate(s) selectively utilized by host microorganisms that confers a health benefit on the host” [190]. The health-promoting properties of synbiotics have been attributed to the potential synergistic actions of their individual components, which comprise both probiotics and prebiotics. In a *Drosophila melanogaster* model of AD, supplementation with a synbiotic, comprising a combination of 3 probiotic strains and total flavonoids of *Laggera alata* powder, reduced Aβ deposition and restored acetylcholinesterase activity, partly due to its combinative action on GABA signaling pathways [191]. In an RCT of 49 older individuals, synbiotic intervention exerted beneficial effects on cognition [192]. Importantly, another double-blind RCT investigated the effects of the fermented milk, kefir, in patients with AD and found that synbiotic supplementation alleviated AD-associated systemic inflammation and improved cognitive deficits [192]. These findings support the use of synbiotics as a dietary approach to improve brain function and modulate the gut microbiota in patients with AD.

### Natural products

Nutritionally derived natural foods have many health benefits that are mainly attributed to their high content of functional macromolecules such as polysaccharides, polyphenols, and bioactive peptides. Indeed, many natural products and bioactive compounds, such as fruits and vegetables, edible mushrooms, medicinal plants, seafood, and green tea, can be obtained through dietary intake, highlighting the potential of the dietary strategy for the treatment of AD.

#### Fruits and vegetables

Fruits and vegetables are whole plant-based foods that are rich in vitamins, flavonoids, minerals, and phytochemicals, which in turn exhibit various biological activities in the host. Many studies have investigated the beneficial effects of fruits such as apples, grapes, mulberries, and blueberries on brain function in AD [193]. In rats with AD, phlorizin, an organic compound generally extracted from apples, has been shown to ameliorate cognitive deficits and neuroinflammation in the brain [194]. Mulberries are rich in bioactive compounds such as polyphenolics and polysaccharides [195]. Importantly, a double-blind RCT reported that blueberry intake improves cognitive function in middle-aged individuals [196]. Moreover, multiple vegetables such as tomatoes, *Capsicum frutescens*, *Monsonia angustifolia*, and cruciferous vegetables have shown potential to prevent the progression of AD.

Dietary supplementation of capsaicin, an agent extracted from hot chili peppers, has been shown to ameliorate behavioral impairments and decrease Aβ and tau deposition in the hippocampus in rats, suggesting a protective role against AD [197]. However, these studies on fruits and vegetables have all been conducted in animals, and no human study has been published until date. As such, future clinical studies should investigate their modulating effects on the gut microbiota to pave the way for dietary strategies involving fruit and vegetable intake for the prevention of AD.

#### Edible mushrooms

Edible mushrooms are the fleshy, edible fruit bodies of several species of macro-fungi, such as *Lentinus subnudus*, *Pleurotus ostreatus*, *Amanita caesarea*, *Agaricus bisporus*, and *Inonotus obliquus* [198]. Mushrooms are the only vegetarian food that can make vitamin D. Because of their high nutritional content and bioactive components (mostly polysaccharides), their use is of interest for health maintenance and disease prevention [199]. Although the mechanisms underlying the neuroprotective and microbiota-modulation functions of edible mushrooms have not been completely understood, their dietary consumption has the potential for preventing the development of AD [200]. In addition, one study reported that polysaccharides isolated from *Inonotus obliquus* and its fruiting bodies via fermentation exhibit neuroprotective effects against AD-like behaviors and that these effects are possibly related to the anti-oxidative and antiapoptotic properties of the bioactive components [201]. Similarly, another polysaccharide extracted from *Amanita caesarea* showed protective effects against the progression of AD [202]. Taken together, these data indicate that edible mushrooms are a potential candidate for the mitigation of AD.

#### Medicinal plants

As there is no complete cure for AD, the global medical profession has focused on medicinal plants, which contain complex active ingredients. Medicinal plants, including Chinese herbs, have been used globally to enhance memory, treat diseases, and improve health. Herbs and medicinal plants are gaining attention for their crucial role in the management of neurological disorders, particularly AD and MCI [203]. *Rhodiola crenulate*, widely used in health foods to treat depression and fatigue, has been shown to have protective effects on cognition in patients with AD [204]. Using pharmacodynamic and urinary metabolomics, the *Schisandra chinensis* polysaccharide has been shown to be associated with AD in rats. Further mechanistic studies have showed that the potential processes associated with its neuroprotective function are the promotion of neurotransmitter production and regulation of endogenous metabolites [205]. Despite our knowledge regarding the neuroprotective function of these herbs in the prevention of AD, there are no published trials examining its clinical effects. Thus, medical plant appears to be a promising AD disease-modifying medical candidate, which deserve to be comprehensively investigated.

#### Other natural foods

In addition to fruits, vegetables, mushrooms, and medicinal plants, there are other naturally occurring products with the potential to reduce AD risk. First, seafood is rich in omega-3 PUFAs, taurine, vitamin D, and selenium, which are essential for the development of brain function [206]. Several studies have demonstrated that sea cucumber (*Cucumaria frondose*), fish, and fish-oil supplementation ameliorate cognitive impairments, suppress neuroinflammation and enhances the microglial/macrophage barrier in different animal models of AD [207, 208]. Moreover, green tea has gained popularity and frequently been referred to as a mood and brain food. Green tea contains numerous phytochemicals and has been shown to benefit human health by increasing mental clarity, improving cognition, and promoting neuromuscular activation and relaxation [209]. Green tea or its polyphenolic extract, epigallocatechin-3-gallate, have been shown to prevent AD-induced learning and memory deficits and inhibit the formation of Aβ aggregates in animal models of AD [[210], [211], [212]]. As for the micronutrients, a recent published review has highlighted the potential effects of selenium-enriched foods and magnesium intervention in alleviating AD [213, 214]. Moreover, using animal models, researchers found that dietary selenium supplementation improved BBB function and cognition [215], and oral administration of magnesium N-Acetyltaurinate enhanced the synaptic plasticity [216].

Given that most published studies on the neuroprotective effects of foods or specific diets have used animal models, further clinical trials are warranted before these findings are translated into dietary guidelines. Because of the need for neuroactive compounds during brain development, further studies should investigate the interactions between dietary components and microbial metabolites via the microbiota–gut–brain axis.

## Challenges and Future Directions

Although there have been promising evidence supporting the contribution of the microbiome to AD, the potential causality and mechanisms are not completely resolved. For example, individuals with enriched Enterobacteriaceae appear to be more susceptible to AD [37], the relative abundance of Bacteroides species is predictive of the development of AD pathologies [217], and varied diet interventions have exhibited effective in alleviating AD-related cognitive decline [218]. Despite decades of hard work to explore the connection between gut microbiome and AD, these observations remain equivocal. In addition, it is important to realize that this research field is highly cross-cutting, such as involving intersections of neuroscience, microbiology, and multi-omics that are influenced by the immune system, lifestyle factor, and genome of the host. Therefore, future research should both identify subpopulation-specific biomarkers to provide optimal treatment and illustrate the underlying mechanisms to propose intervention strategy. Advances in humanized mouse models, genomic technologies, and bioinformatic tools have made it possible to explore the underlying mechanisms and enable the development of microbiome-targeted manipulation options.

Given that clinical therapeutic approaches have not yet proved effective in halting the development of AD, a successful intervention strategy would both prevent cognitive decline in the population at a high risk of AD and improve the cognition at early-onset of AD formation (individuals with MCI). Although preclinical studies using animal models open the possibility of microbiome-based diet intervention, there are still a lot of challenges to translate laboratory discoveries to clinic. First, most dietary interventions need to be continuous and take a long period, making it difficult to maintain compliance. Second, the beneficial effects of a specific dietary intervention might be less pronounced than expected because of the resilience and dynamics of the human gut microbiome. Another major challenge is that whether the microbiome-based intervention strategy for AD can be generalized or need to be personalized. Furthermore, most interventional studies mainly focused on the effectiveness, ignoring the fact that many factors, including dosage, disease status, and inflammatory state, influence the function of intervention. Thus, extensive, iterative studies in well-designed clinical trials are required to be conduct in academic settings supported by government or foundation. Given that human studies are just starting to emerge, certain methodological factors of experimental design should be included to ensure the quality and stability of results. For example, the dose-dependent association between specific nutrients or metabolites and AD, the safety and effectiveness of live probiotics, and the disease severity of the included subgroup population should be taken into consideration.

## Conclusions and Perspectives

Historically, the gut microbiota, microbial metabolites, and brain have been studied independently. However, the idea that the gut microbiota-driven barrier and BBB dysfunction underlie various neurological disorders has encouraged closer inspection of these functional changes for the modulation of microbiota–metabolite–brain interactions. Identifying and characterizing the causal or contributing roles of specific microbiome components and microbial metabolites in AD can facilitate transformative advances in therapeutic strategies. Dietary nutrients and specific bioactive components that affect brain function and regulate microbiota composition, both individually and together as part of a diet, have the potential to delay the progression of AD. Thus, dietary interventions and microbiome-targeted manipulation strategies are gaining more attention for the prevention of cognitive impairments and AD progression.

Despite our increased awareness of the contribution of the microbiota and its metabolites to the progression of AD, future efforts should focus on causality and potential mechanisms. In terms of the gut microbial metabolites in AD, previous studies have largely described alterations in specific metabolites correlated with disease states, whereas the effects of specific gut-derived metabolites on brain function and behavior have rarely been studied. Importantly, a deeper understanding of the pathways mediating gut–microbiota–brain connections in AD may lead the way for novel microbiome-targeted dietary interventions for the prevention of AD. However, most studies have explored the beneficial effects of various dietary nutrients or components in animal models, and well-designed clinical and translational studies aimed at improving brain health and preventing the progression of AD are lacking. Taken together, the data presented herein indicate that the microbiota and their metabolites provide attractive targets for promoting brain function by either targeting the microbial community or by supplementation with specific dietary components.

## Funding

Supported by the National Natural Science Foundation of China (No. 31972052, 32021005, 31820103010), the Fundamental Research Funds for the Central Universities (JUSRP22006, JUSRP51501), the Program of Collaborative Innovation Centre of Food Safety and Quality Control in Jiangsu Province.

## Author disclosures

The authors report no conflicts of interest.
